# Bedside risk score for medical therapy failure in small‐volume BPH: Temporal validation

**DOI:** 10.1002/bco2.70217

**Published:** 2026-04-29

**Authors:** Numan Alam, Naveed Ahmad, Syed Hamzah Shirazi, Aftab Ahmed, Bilal Suria, Jamshid Ali, Muhammad Anas Ghazi, Elham Shenawa, Mirza Muhammad Hadeed Khawar

**Affiliations:** ^1^ DHQ Teaching Hospital Mardan Pakistan; ^2^ Alkhidmat Raazi Hospital Rawalpindi Pakistan; ^3^ Sheikh Khalifa Bin Zaid/CMH Hospital Muzaffarabad Pakistan; ^4^ Al‐Tibri Medical College & Hospital Isra University Karachi Pakistan; ^5^ THQ Hospital Banda Daud Shah Pakistan; ^6^ North Manchester General Hospital Manchester UK; ^7^ Faculty of Medicine Balkh University Mazar‐i‐Sharif Afghanistan; ^8^ Services Institute of Medical Sciences Lahore Pakistan

**Keywords:** bedside risk score, benign prostatic hyperplasia, diabetes mellitus, Intravesical prostatic protrusion, medical therapy failure, small prostate volume, temporal validation

## Abstract

**Purpose:**

Medical therapy is the first‐line treatment for lower urinary tract symptoms (LUTS) secondary to benign prostatic hyperplasia (BPH). However, predictors of treatment failure in men with small prostate volume (<30 ml) remain poorly defined. This study aimed to develop and temporally validate a simple bedside risk score for predicting medical therapy failure in this specific subgroup.

**Methods:**

We performed a retrospective cohort study of 201 men aged ≥50 years with IPSS ≥8 and prostate volume <30 ml who started medical therapy between 2015 and 2025. Treatment failure was defined as surgical intervention, acute urinary retention or IPSS worsening by ≥4 points. Independent predictors were identified using multivariable logistic regression. A practical integer risk score was derived from the strongest predictors. Temporal validation was conducted by splitting the cohort chronologically (derivation set 2015–2020, *n* = 120; validation set 2021–2025, *n* = 81).

**Results:**

During a median follow‐up of 24 months, 66 patients (32.8%) experienced treatment failure. Independent predictors included higher IPSS, greater BPH Impact Index, increased intravesical prostatic protrusion, lower maximum flow rate, higher post‐void residual volume and diabetes mellitus. The bedside risk score stratified patients into low‐risk (0–3 points: 11.0% failure), moderate‐risk (4–7 points: 32.9%) and high‐risk (8–13 points: 77.5%) categories. The model demonstrated good discrimination (AUC 0.789; bootstrap‐corrected 0.782) and maintained strong performance in temporal validation (derivation AUC 0.799; validation AUC 0.821).

**Conclusion:**

This novel bedside risk score reliably predicts medical therapy failure in small‐volume BPH using readily available clinical parameters. It may enable early risk stratification and timely intervention, particularly in populations with high diabetes prevalence.

## 1 INTRODUCTION

Benign prostatic hyperplasia (BPH) is a common condition in aging men and a leading cause of lower urinary tract symptoms (LUTS) that impair quality of life. Histological evidence of BPH is found in >50% of men aged 50–60 years and approaches 80% by age 80.^1^, ^2^ In South Asia, community surveys report LUTS prevalence exceeding 30% in adult males, closely linked to advancing age and rising metabolic disorders.^3^, ^4^

International guidelines from the American Urological Association and European Association of Urology recommend medical therapy as first‐line treatment for moderate‐to‐severe LUTS.^5^, ^6^ Alpha‐adrenergic blockers form the mainstay, with 5‐alpha reductase inhibitors or antimuscarinics added according to prostate size and symptom profile. Although effective for many patients, pharmacological treatment fails in 20–40% of cases, resulting in acute urinary retention, symptom worsening or surgical intervention.^7^, ^8^ Identifying predictors of failure is therefore critical for timely risk stratification and avoidance of unnecessary morbidity.

Most data on BPH progression derive from cohorts with moderate‐to‐large prostates (>30 ml), in which static obstruction from glandular enlargement predominates.^9^, ^10^, ^11^ Landmark trials such as MTOPS and CombAT linked larger prostate volume, elevated PSA and severe baseline IPSS to increased progression risk. However, these findings may not apply to the 20–30% of men with small prostate volumes (<30 ml).^12^, ^13^ In this subgroup, symptoms frequently stem from dynamic factors—including intravesical prostatic protrusion (IPP), prostatic urethral angulation, detrusor overactivity and reduced bladder contractility—rather than volumetric enlargement alone.

Metabolic comorbidities, particularly diabetes mellitus, appear to exacerbate LUTS in small prostates through neuropathic and microvascular effects on detrusor function.^14^, ^15^ Despite its clinical relevance, predictors of medical therapy failure in small‐volume BPH remain inadequately defined. Existing studies are limited, predominantly from East Asian or Western populations and yield inconsistent results regarding IPP, post‐void residual volume and diabetes.^16^, ^17^, ^18^ Dedicated analyses from South Asian cohorts—where adult diabetes prevalence reaches 14–20%—are especially scarce.

To our knowledge, this is one of the largest dedicated studies focusing exclusively on men with prostate volume <30 ml, a common yet distinctly understudied phenotype that accounts for 20–30% of all BPH cases but has been systematically excluded or diluted in landmark progression trials and recent international guidelines. By concentrating on this specific subgroup within a South Asian population with high diabetes prevalence, the present study aimed to clarify the dominant mechanisms of medical therapy resistance and to develop a simple, practical risk score that can be applied at the bedside for rapid risk stratification and more individualised decision‐making.

## 2 METHODS

### 2.1 Study design and ethical considerations

This retrospective cohort study examined the medical records of men who received initial medical treatment for lower urinary tract symptoms at the Department of Urology, District Headquarters Teaching Hospital Mardan, Pakistan. We identified consecutive eligible patients treated between January 2015 and December 2025, with final data verification and statistical analysis completed in early 2026. The institutional Ethics Review Board approved the protocol (Approval Number 13195/MS/Supdt, dated 10 December 2025) and waived the need for informed consent because of the retrospective nature of the work. The study was conducted in accordance with the Declaration of Helsinki and reported according to the STROBE guidelines for observational cohort studies.

### 2.2 Patient selection

**FIGURE 1:**
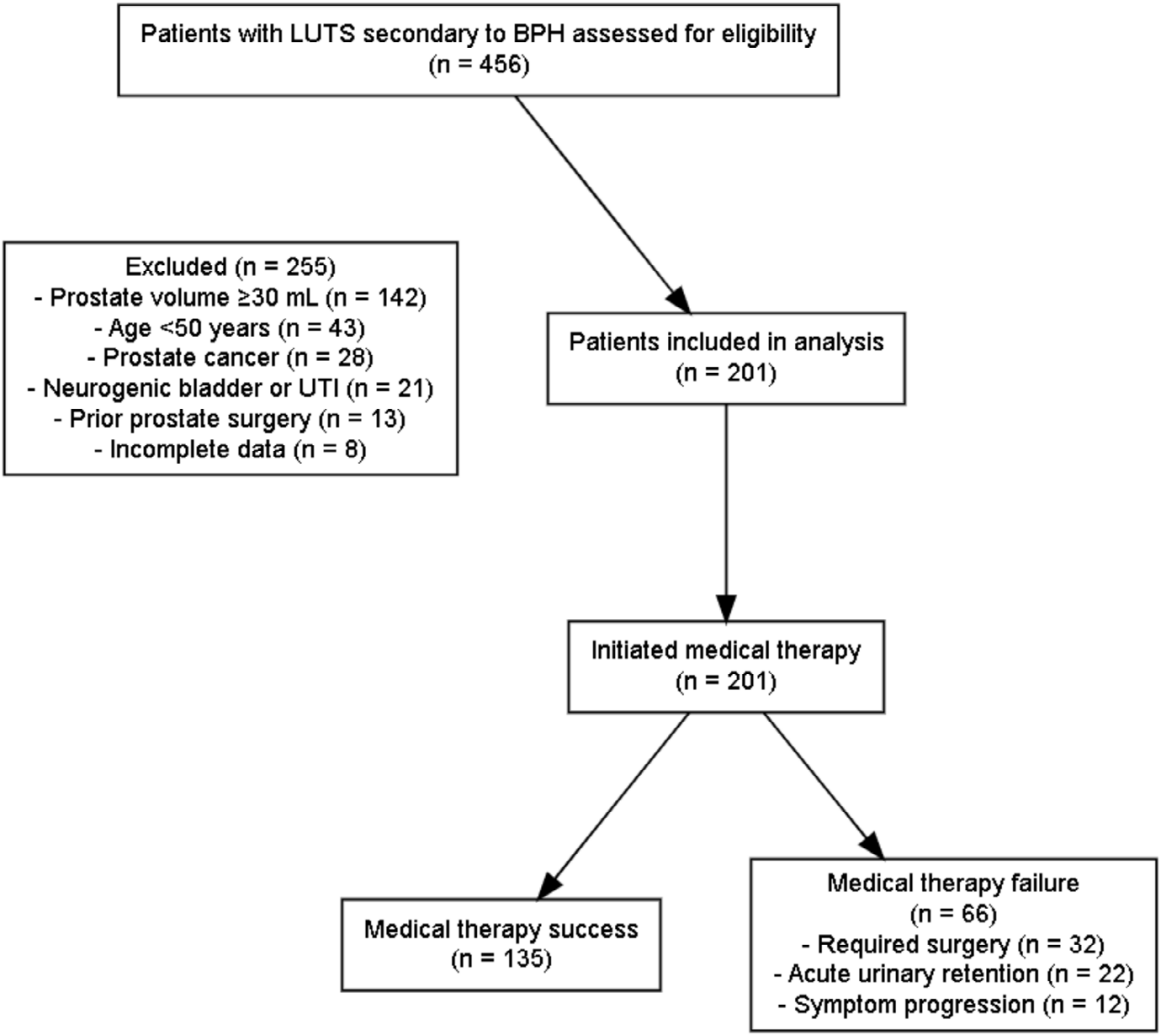
Patient selection and study flow. Study flow diagram illustrating patient selection, inclusion and exclusion criteria, and final outcomes in the retrospective cohort of men with small prostate volume and lower urinary tract symptoms (LUTS) treated with medical therapy.

We included men aged 50 years or older who presented with lower urinary tract symptoms that were clinically attributed to benign prostatic hyperplasia after assessment of symptom history, digital rectal examination, prostate imaging and exclusion of alternative diagnoses. All participants had a baseline International Prostate Symptom Score of 8 or higher and a total prostate volume below 30 ml. Prostate volume was measured by transrectal ultrasonography using the standard ellipsoid formula (length × width × height × 0.52); when magnetic resonance imaging had been performed, the radiologist‐reported volume was used. Patients were excluded if they had known or suspected prostate cancer, neurogenic bladder dysfunction, active urinary tract infection at presentation, previous prostate surgery, prostate volume of 30 ml or greater, immediate surgical intervention without a trial of medical therapy or follow‐up shorter than 12 months. Detailed reasons for exclusion are shown in Figure 1.

### 2.3 Baseline assessment

At the initiation of medical therapy we recorded age in years, body mass index in kg/m^2^ and duration of symptoms in months. Symptom severity was evaluated with the International Prostate Symptom Score (0–35), from which storage and voiding subscores were derived, together with the IPSS quality‐of‐life question (0–6) and the BPH Impact Index (0–13). Anatomical measurements obtained by transrectal ultrasonography included total prostate volume in millilitres, intravesical prostatic protrusion (perpendicular distance from the protrusion tip to the bladder neck in the sagittal plane) in millimetres and prostatic urethral angle in degrees. Functional parameters comprised maximum urinary flow rate in ml/s measured by uroflowmetry and post‐void residual urine volume in ml determined by ultrasound within 10 min after voiding. Serum prostate‐specific antigen was recorded in ng/ml. Comorbidities were defined as follows: diabetes mellitus according to American Diabetes Association criteria, hypertension by documented diagnosis or use of antihypertensive medication, and renal impairment as an estimated glomerular filtration rate below 60 ml/min/1.73 m^2^. Smoking status was categorised as never, former or current smoker.

### 2.4 Medical therapy protocol

All patients started treatment with tamsulosin 0.4 mg once daily. In those with predominant storage symptoms, solifenacin 5 mg once daily was added at the discretion of the treating urologist. Finasteride 5 mg daily was prescribed selectively when prostate‐specific antigen exceeded 1.5 ng/ml or when symptom response to alpha‐blocker therapy remained inadequate. Patients were classified as receiving either alpha‐blocker monotherapy or combination therapy. Adherence was assessed through prescription refill records, with adequate adherence defined as filling more than 80% of prescribed doses.

### 2.5 Follow‐up and outcome definition

Routine follow‐up visits were scheduled at 3, 6, 12 and 24 months after treatment initiation, with annual assessments thereafter as clinically appropriate. Medical therapy failure, the primary outcome, was defined as the occurrence of any of the following: surgical intervention (transurethral resection of the prostate or laser enucleation), acute urinary retention requiring catheterisation or a clinically meaningful increase in IPSS of 4 points or more from baseline. Time to failure was calculated from the date of treatment start to the first documented event; patients without failure were censored at the date of their last recorded visit.

### 2.6 Urodynamic evaluation

Non‐invasive uroflowmetry and post‐void residual urine measurement were performed in every patient at baseline. Multichannel invasive urodynamic studies were undertaken selectively when diagnostic uncertainty persisted or symptoms failed to improve with initial treatment.

### 2.7 Statistical analysis

Analyses were performed using R Software Version 4.5.1 (R Foundation for Statistical Computing, Vienna, Austria). Continuous variables are summarised as mean with standard deviation or median with interquartile range according to their distribution and categorical variables as frequencies and percentages. Between‐group comparisons used independent *t* tests or Mann–Whitney *U* tests for continuous data and chi‐square or Fisher's exact tests for categorical data, as appropriate. Variables associated with the outcome at *p* < 0.10 in univariable analysis were entered into multivariable logistic regression models adjusted for age, major comorbidities and treatment category. Time‐to‐event data were analysed with Kaplan–Meier curves and Cox proportional hazards regression, with the proportional hazard assumption confirmed by Schoenfeld residuals.

The final multivariable logistic regression model was developed on the derivation cohort. Variance inflation factors (VIF) were calculated to assess multicollinearity; all VIF values were <2.5, indicating no substantial collinearity. Missing data were minimal (<3% for key variables) and handled by complete‐case analysis. Model discrimination was assessed by the area under the receiver operating characteristic curve (AUC), with internal validation performed by 1000 bootstrap resamples. Decision curve analysis was performed to evaluate the clinical utility of the prediction model across a range of threshold probabilities. Net benefit was calculated for each threshold as the difference between the proportion of true positives and the weighted proportion of false positives, with weighting defined by the threshold odds (*Pt*/[1 − *Pt*]). The full multivariable model was compared with models containing individual predictors and the two reference strategies of treating all patients (‘predicting all’) or treating none (‘predicting none’).

A pre‐specified sensitivity analysis was performed using only hard clinical endpoints (surgical intervention or acute urinary retention; 54 events), excluding patients whose failure was defined solely by an IPSS increase of ≥4 points. The same multivariable model was refitted on this hard‐endpoint outcome to assess whether discriminatory performance remained stable.

To facilitate bedside application, a simple integer risk score was derived from the five strongest and most clinically accessible predictors retained after accounting for collinearity. The integer bedside risk score was created by dichotomising these predictors at clinically meaningful cutoffs (optimised by Youden index or standard thresholds) and assigning points proportionally to each predictor's regression coefficient (*β*) from the final model, rounded to the nearest integer for bedside simplicity (exact *β* coefficients, odds ratios and point‐assignment details are provided in Table S1). BPH Impact Index was excluded from the simplified score because of high correlation with total IPSS (Spearman *ρ* = 0.78).

Temporal validation was performed by chronological cohort splitting (derivation set: patients treated 2015–2020, *n* = 120; validation set: patients treated 2021–2025, *n* = 81). The final multivariable model was fitted on the derivation cohort and applied to the validation cohort without refitting. A two‐sided *p* value below 0.05 was considered statistically significant.

## 3 RESULTS

### 3.1 Patient cohort and follow‐up

Of 456 men screened, 201 satisfied the inclusion criteria and formed the study cohort (Figure 1). Median follow‐up was 24 months (interquartile range 12–36). Medical therapy failure developed in 66 patients (32.8%) at a median of 18 months (interquartile range 9–27). The failure events comprised surgical intervention in 32 patients (48.5%), acute urinary retention in 22 (33.3%) and clinically meaningful symptom worsening (IPSS increase ≥4 points) in 12 (18.2%).

### 3.2 Baseline characteristics

Patients who experienced failure were older (68.5 ± 8.0 vs. 64.4 ± 7.4 years, *p* < 0.001) and carried a heavier symptom burden at presentation (Table 1). Mean total IPSS was 21.6 ± 5.7 in the failure group compared with 14.2 ± 4.7 in the success group (*p* < 0.001). BPH Impact Index scores were correspondingly higher (7.4 ± 3.1 vs. 4.9 ± 2.0, *p* < 0.001). Anatomical and functional markers of obstruction were also worse among those who later failed: intravesical prostatic protrusion measured 8.0 ± 3.4 mm versus 5.1 ± 2.7 mm (*p* < 0.001), maximum urinary flow rate was lower (7.4 ± 3.0 vs. 12.0 ± 3.6 ml/s, *p* < 0.001) and post‐void residual urine volume was substantially greater (126.7 ± 47.2 vs. 47.2 ± 28.5 ml, *p* < 0.001). Total prostate volume did not differ meaningfully between groups (21.1 ± 4.2 vs. 22.2 ± 4.0 ml, *p* = 0.062). Diabetes mellitus was far more prevalent in the failure group (51.5% vs. 20.0%, *p* < 0.001), as were hypertension and renal impairment.

**TABLE 1:** Baseline characteristics of participants stratified by medical therapy outcome.

Characteristic	Overall (*n* = 201)	Success (*n* = 135)	Failure (*n* = 66)	*p* value
Demographics				
Age, years	65.7 ± 7.8	64.4 ± 7.4	68.5 ± 8.0	<0.001
Body mass index, kg/m^2^	26.6 ± 3.9	26.4 ± 3.9	27.0 ± 3.8	0.301
Duration of symptoms, months	21.3 ± 14.1	17.5 ± 11.2	29.1 ± 16.4	<0.001
Symptom measures				
IPSS total score (0–35)	16.6 ± 6.1	14.2 ± 4.7	21.6 ± 5.7	<0.001
IPSS storage subscore	6.5 ± 3.3	5.5 ± 3.1	8.6 ± 2.8	<0.001
IPSS voiding subscore	10.1 ± 5.9	8.7 ± 5.2	12.9 ± 5.6	<0.001
IPSS quality‐of‐life score (0–6)	2.9 ± 1.0	2.7 ± 1.0	3.4 ± 1.0	<0.001
BPH Impact Index (0–13)	5.7 ± 2.8	4.9 ± 2.0	7.4 ± 3.1	<0.001
Anatomical parameters				
Total prostate volume, mL	21.8 ± 4.1	22.2 ± 4.0	21.1 ± 4.2	0.062
Intravesical prostatic protrusion, mm	6.1 ± 3.3	5.1 ± 2.7	8.0 ± 3.4	<0.001
Prostatic urethral angle, degrees	33.5 ± 14.2	29.8 ± 11.0	40.7 ± 16.2	<0.001
Functional parameters				
Maximum urinary flow rate (Qmax), ml/s	10.4 ± 4.1	12.0 ± 3.6	7.4 ± 3.0	<0.001
Post‐void residual urine volume (PVR), ml	74.3 ± 52.6	47.2 ± 28.5	126.7 ± 47.2	<0.001
Prostate‐specific antigen, ng/ml	2.9 ± 1.7	2.5 ± 1.4	3.6 ± 2.0	<0.001
Comorbidities				
Diabetes mellitus, *n* (%)	61 (30.3)	27 (20.0)	34 (51.5)	<0.001
Hypertension, *n* (%)	107 (53.2)	63 (46.7)	44 (66.7)	0.012
Renal impairment, *n* (%)	29 (14.4)	14 (10.4)	15 (22.7)	0.033
Smoking status, *n* (%)				0.011
Never	82 (40.8)	63 (46.7)	19 (28.8)	
Former	72 (35.8)	48 (35.6)	24 (36.4)	
Current	47 (23.4)	24 (17.8)	23 (34.8)	

*Note*: Data are presented as mean ± standard deviation for continuous variables and number (percentage) for categorical variables. *p* values were derived using independent *t* tests or Mann–Whitney *U* tests for continuous variables and chi‐square or Fisher's exact tests for categorical variables, as appropriate. Mean ± standard deviation is reported for approximately normally distributed variables (age, IPSS, BPH Impact Index, Qmax); median (interquartile range) was used for skewed variables (duration of symptoms, post‐void residual urine volume) in sensitivity analyses, with identical conclusions.

Abbreviations: BPH, benign prostatic hyperplasia; IPSS, International Prostate Symptom Score; PVR, post‐void residual urine volume; Qmax, maximum urinary flow rate.

### 3.3 Medical therapy exposure

Alpha‐blocker monotherapy was prescribed to 148 men (73.6%), whereas 53 (26.4%) received combination therapy. The distribution of treatment regimens did not differ between success and failure groups (*p* = 0.41), and treatment category was not independently associated with outcome after multivariable adjustment.

### 3.4 Functional outcomes

Men whose therapy succeeded showed clear improvement by last follow‐up: mean IPSS decreased by 4.8 ± 3.2 points, maximum flow rate increased by 3.1 ± 2.0 ml/s and post‐void residual volume fell by 23.3 ± 13.0 ml (Table 2). In contrast, the failure group experienced worsening: IPSS rose by 1.6 ± 4.4 points, flow rate declined by 0.7 ± 2.2 ml/s and residual volume increased by 29.9 ± 19.5 ml (all *p* < 0.001).

**TABLE 2:** Clinical outcomes stratified by medical therapy group.

Outcome	Success (*n* = 135)	Failure (*n* = 66)	*p* value
Symptom change from baseline			
Change in IPSS total score	−4.8 ± 3.2	+1.6 ± 4.4	<0.001
Functional change from baseline			
Change in maximum urinary flow rate (Qmax), ml/s	+3.1 ± 2.0	−0.7 ± 2.2	<0.001
Change in post‐void residual urine volume (PVR), ml	−23.3 ± 13.0	+29.9 ± 19.5	<0.001
Clinical events during follow‐up			
Acute urinary retention, *n* (%)	5 (3.7%)	34 (51.5%)	<0.001
Surgical intervention, *n* (%)	0 (0%)	32 (48.5%)	—

*Note*: Data are presented as mean ± standard deviation for continuous variables and number (percentage) for categorical variables. *p* values were derived from independent *t* tests for continuous variables and chi‐square tests for categorical variables. Mean ± standard deviation is reported for approximately normally distributed variables (IPSS change and Qmax change); median (interquartile range) was used for skewed variables (PVR change) in sensitivity analyses, with identical conclusions. Five patients in the success group experienced a single episode of acute urinary retention that resolved spontaneously with conservative catheterisation and did not meet the study definition of treatment failure (requirement for ongoing catheterisation or surgical intervention).

Abbreviations: IPSS, International Prostate Symptom Score; PVR, post‐void residual urine volume; Qmax, maximum urinary flow rate.

### 3.5 Time‐to‐failure analysis

**FIGURE 2:**
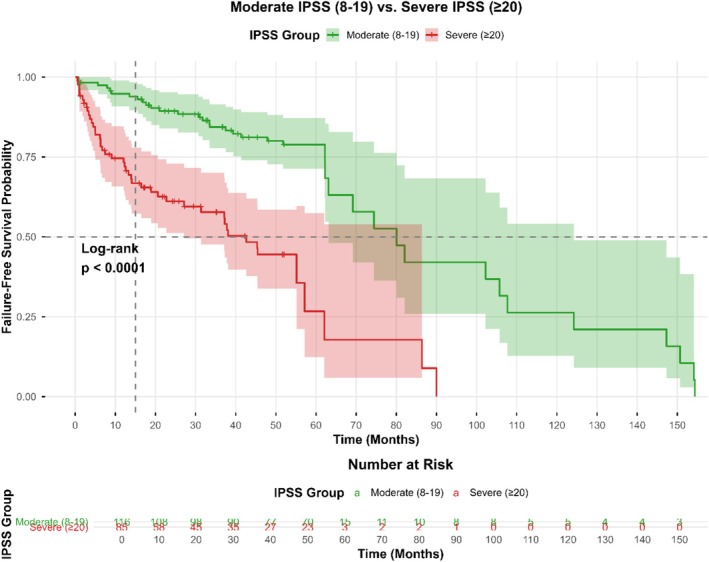
Failure‐free survival according to baseline symptom severity. Kaplan–Meier estimates of failure‐free survival in men with small‐volume benign prostatic hyperplasia (*n* = 201), stratified by baseline International Prostate Symptom Score (IPSS): moderate (8–19; *n* = 116) versus severe (≥20; *n* = 85). Shaded areas represent 95% confidence intervals. Median failure‐free survival was 30 months (95% CI 27–33) in the moderate group and 15 months (95% CI 12–18) in the severe group. Log‐rank *p* < 0.0001. Numbers at risk are shown at predefined time intervals below the *x* axis.

Kaplan–Meier curves demonstrated markedly shorter failure‐free survival among patients with severe baseline symptoms (IPSS ≥ 20) compared with those who had moderate symptoms (log‐rank *p* < 0.001; Figure 2). Median failure‐free survival was 15 months (95% CI 12–18) in the severe group versus 30 months (95% CI 27–33) in the moderate group.

### 3.6 Multivariable predictors of therapy failure

**FIGURE 3:**
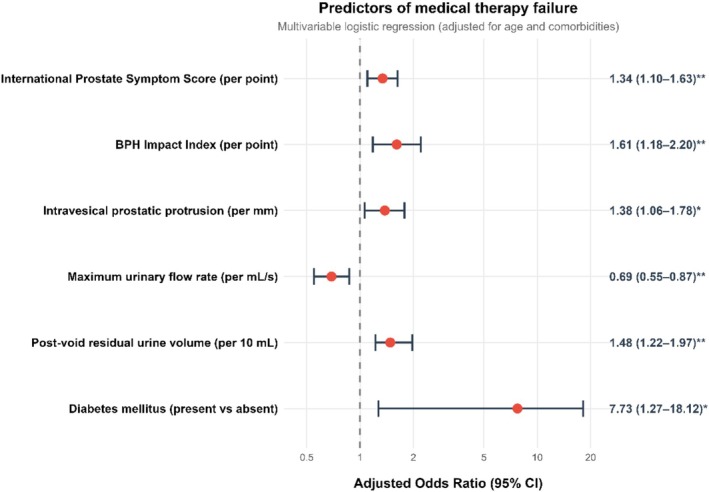
Predictors of medical therapy failure. Forest plot displaying odds ratios (ORs) with 95% confidence intervals from the multivariable logistic regression analysis identifying baseline predictors of medical therapy failure, adjusted for age and comorbidities. The dashed vertical line indicates the null effect (OR = 1). Statistical significance is denoted by asterisks.

After adjustment for age, comorbidities and treatment category, six baseline variables remained independently associated with medical therapy failure (Figure 3): higher IPSS (odds ratio 1.34 per point, 95% CI 1.10–1.63, *p* = 0.004), higher BPH Impact Index (odds ratio 1.61 per point, 95% CI 1.18–2.20, *p* = 0.003), greater intravesical prostatic protrusion (odds ratio 1.38 per mm, 95% CI 1.06–1.78, *p* = 0.015), lower maximum urinary flow rate (odds ratio 0.69 per ml/s, 95% CI 0.55–0.87, *p* = 0.002), higher post‐void residual urine volume (odds ratio 1.04 per ml, 95% CI 1.02–1.07, *p* < 0.001) and presence of diabetes mellitus (odds ratio 7.73, 95% CI 2.71–22.05, *p* < 0.001). Prostate‐specific antigen lost significance after multivariable adjustment (*p* = 0.082). Prostatic urethral angle, although significantly higher in the failure group on univariable analysis, did not remain independently predictive after multivariable adjustment (*p* = 0.12). To facilitate bedside application, we derived a simple integer risk score from these predictors (Table 3). The score stratified patients into low‐, moderate‐ and high‐risk categories with observed failure rates of 11.0%, 32.9% and 77.5%, respectively (*p* for trend < 0.001). The robustness of these findings was confirmed in sensitivity analyses (Figure S1).

**TABLE 3:** Integer‐based clinical risk score for predicting medical therapy failure in small‐volume BPH (*n* = 201).

A. Scoring system derived from multivariable model		
Predictor	Clinical definition	Points assigned
Diabetes mellitus	Present (ADA criteria)	4
IPSS ≥ 20	Severe symptom category	3
Intravesical prostatic protrusion ≥8 mm	TRUS measurement	2
Maximum urinary flow rate (Qmax) <8 ml/s	Uroflowmetry	2
Post‐void residual urine volume ≥100 ml	Ultrasound measurement	2

B. Risk stratification and observed outcomes				
Risk category	Score range	Patients (*n*, %)	Failures (*n*)	Observed failure rate
Low risk	0–3	82 (40.8%)	9	11.0%
Moderate risk	4–7	79 (39.3%)	26	32.9%
High risk	8–13	40 (19.9%)	31	77.5%

C. Predictive performance of risk score			
Area under the ROC curve (AUC): 0.76 (95% CI 0.68–0.83) Optimal cutoff ≥5 points: ○ Sensitivity: 74% ○ Specificity: 68%			

*Note*: Total possible score: 0–13. *p* for trend across risk categories: <0.001. Points were assigned by dividing each predictor's regression coefficient (*β*) from the final multivariable model by the smallest absolute *β* and rounding to the nearest integer. Exact *β* coefficients, odds ratios and full derivation details are provided in Table S1. BPH Impact Index was excluded from the simplified score because of collinearity with total IPSS.

### 3.7 Model performance

**FIGURE 4:**
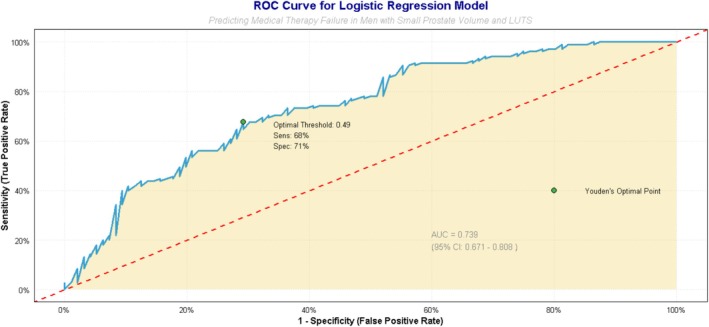
Discriminatory performance of the prediction model. Receiver operating characteristic (ROC) curve evaluating the ability of the multivariable logistic regression model to predict medical therapy failure. The area under the curve (AUC) is 0.789 (95% CI 0.725–0.853). The optimal Youden's index threshold, sensitivity and specificity are annotated.

The multivariable model demonstrated good discriminatory ability, with an area under the receiver operating characteristic curve of 0.789 (95% CI 0.725–0.853; Figure 4). Internal validation by 1000 bootstrap resamples yielded a bias‐corrected AUC of 0.782. The bias‐corrected calibration slope was 0.89 (95% CI 0.72–1.06) with an intercept of 0.03, indicating minimal overfitting, and the Brier score was 0.184 (Figure S2). At the optimal Youden threshold of 0.49, sensitivity was 68% and specificity 71%. The model retained good discriminatory performance in temporal validation (Figure S3): AUC 0.799 (95% CI 0.718–0.880) in the derivation cohort (2015–2020) and AUC 0.821 (95% CI 0.731–0.911) in the independent temporal validation cohort (2021–2025), demonstrating acceptable generalisability over time. In a pre‐specified sensitivity analysis restricted to hard clinical endpoints (surgical intervention or acute urinary retention only; 54 events), the model maintained comparable discriminatory performance, with an AUC of 0.782 (95% CI 0.711–0.853) versus 0.789 for the primary composite outcome (Figure S5), confirming that the identified predictors remain robust when softer symptom‐based outcomes are excluded.

### 3.8 Clinical utility

Decision curve analysis demonstrated that the full multivariable model provided superior net clinical benefit compared with single‐predictor models and the default strategies of treating all or no patients across the clinically relevant threshold probability range of 20–60% (Figure S4). The full model consistently outperformed models based on any single variable (IPSS, diabetes, intravesical prostatic protrusion or maximum flow rate), confirming the added value of integrating symptom severity, dynamic obstruction parameters and metabolic comorbidity.

## 4 DISCUSSION

In this cohort of 201 men with lower urinary tract symptoms and prostate volumes below 30 ml, medical therapy failed in nearly one third of patients over a median follow‐up of 24 months. Failure was independently predicted by greater baseline symptom severity, higher BPH Impact Index, more pronounced intravesical prostatic protrusion, lower maximum flow rate, elevated post‐void residual volume and the presence of diabetes mellitus. Total prostate volume itself carried no predictive value within this strictly defined small‐gland subgroup. Temporal validation confirmed stable discriminatory performance over time, supporting the robustness of the model in a real‐world South Asian setting. Furthermore, model performance remained virtually unchanged when the analysis was restricted to hard clinical endpoints only, reinforcing the robustness of symptom severity, intravesical prostatic protrusion, post‐void residual volume and diabetes as predictors of clinically meaningful progression.

These results highlight a distinct pathophysiology in small‐volume benign prostatic hyperplasia. Landmark trials such as MTOPS and CombAT established the importance of prostate enlargement and elevated PSA in disease progression,^7^, ^8^ yet those studies largely enrolled men with glands larger than 30 ml.^11^, ^12^ In the present series, dynamic obstruction from intravesical protrusion and functional impairment of bladder emptying emerged as the dominant drivers of treatment resistance, even when overall gland size remained modest.^14^, ^16^ The strong association with diabetes mellitus aligns with growing evidence that chronic hyperglycaemia impairs detrusor contractility through neuropathy and microvascular damage.^17^, ^18^ In our population, where diabetes prevalence reaches 14–20%, this comorbidity appears particularly consequential^4^ and may explain why alpha‐blockade alone frequently proved insufficient. In this regard, broader public health challenges such as the recent global malnutrition crisis exacerbated by disruptions in international aid (including USAID withdrawal) further threaten nutritional status and metabolic health in low‐ and middle‐income countries such as Pakistan, potentially compounding the risk of medical therapy failure in vulnerable BPH populations.^19^, ^20^

The simple integer risk score derived from these predictors offers a practical bedside tool. Men scoring 8 or higher faced a 77.5% failure rate, whereas those with scores of 0–3 experienced failure in only 11%. Such stratification could guide clinicians towards closer surveillance or earlier consideration of surgical options, especially in resource‐limited settings where prolonged ineffective medical therapy carries both clinical and economic costs. Decision curve analysis further supported the practical usefulness of the derived risk score. The full model achieved higher net clinical benefit than default strategies across the most plausible range of threshold probabilities (20–60%), indicating that implementation of the bedside score would improve patient selection for early surgical intervention while avoiding overtreatment in low‐risk individuals. This is particularly relevant in South Asian populations with high diabetes prevalence, where accurate risk stratification can reduce unnecessary morbidity and optimise healthcare resources. The calibration slope close to 1 after bootstrap correction confirms that predicted probabilities are reliable for bedside use. This supports the clinical utility of the simple risk score derived from this model. Integration of this simple bedside score into routine outpatient electronic health records or patient‐facing mobile decision‐support tools could further facilitate real‐time risk stratification and shared decision‐making at the point of care.

Several limitations must be acknowledged. The single‐centre retrospective design in Pakistan introduces risks of selection and information bias, and treatment decisions (including addition of solifenacin or finasteride) reflected routine clinical practice rather than a fully standardised protocol. Medication adherence was assessed indirectly via prescription refill records, which may overestimate true compliance. Intravesical prostatic protrusion and prostatic urethral angle measurements by transrectal ultrasonography are operator‐dependent; inter‐observer reliability could not be quantified in this retrospective study. Invasive urodynamic studies were performed only selectively, limiting our ability to fully evaluate the role of detrusor underactivity across the entire cohort. Although the composite outcome included a softer symptom‐based component (IPSS increase ≥4 points in 18% of failures), sensitivity analysis restricted to hard clinical endpoints (surgery or acute urinary retention) yielded nearly identical discriminatory performance. Finally, although temporal validation within the same institution showed stable performance, true external validation in independent cohorts from different geographic, ethnic and healthcare settings is essential before widespread clinical adoption. Additionally, we did not account for long‐term exposure to fine particulate matter (PM2.5), which has been strongly linked to increased cardiovascular risk in large meta‐analyses and may act as an unmeasured environmental modifier of metabolic and urological outcomes in our South Asian cohort.^21^

Nevertheless, this study addresses a clinically important gap. By restricting inclusion to men with prostate volumes below 30 ml and focusing on a South Asian cohort with high metabolic comorbidity, we provide data that complement and extend findings from East Asian and Western series.^22^, ^23^ Although temporal validation demonstrated good generalisability within our setting, prospective multicentre external validation—ideally across different ethnic groups and healthcare systems with standardised measurement protocols—is essential before widespread clinical adoption of this risk score.

## 5 CONCLUSION

Among men with lower urinary tract symptoms and small prostate volume, baseline symptom severity, dynamic obstruction parameters and diabetes mellitus independently predict failure of medical therapy. A simple risk score based on these factors stratifies patients effectively and may assist clinicians in timely decision‐making. Prospective multicentre external validation in diverse populations and healthcare settings is warranted before routine clinical implementation of this bedside risk score.

## AUTHORS CONTRIBUTIONS

Numan Alam conceived and designed the study and supervised data collection. Naveed Ahmad and Syed Hamzah Shirazi contributed to the study design, clinical interpretation and drafting of the manuscript. Aftab Ahmed, Bilal Suria and Jamshid Ali performed data acquisition and verification. Muhammad Anas Ghazi provided methodological and statistical expertise and critically revised the manuscript for important intellectual content. Elham Shenawa contributed to study coordination, manuscript editing and final approval of the version to be submitted. Mirza Muhammad Hadeed Khawar provided supervision. All authors read and approved the final manuscript.

## CONFLICT OF INTEREST STATEMENT

The authors declare no conflicts of interest.

## Supporting information


**Figure S1.** Sensitivity analyses demonstrating robustness of independent predictors and model discrimination for predicting medical therapy failure in men with small prostate volume (<30 ml). Panel A shows the odds ratio for diabetes mellitus in the main model and monotherapy‐only sensitivity analysis. Panel B shows the area under the receiver operating characteristic curve (AUC) across three analytical subsets.
**Figure S2.** Calibration plot of the final multivariable logistic regression model for predicting medical therapy failure in men with small‐volume benign prostatic hyperplasia (n = 201). The apparent (dotted) and bias‐corrected (solid) curves were obtained from 1000 bootstrap resamples. The dashed line represents perfect calibration (slope = 1, intercept = 0). Mean absolute error = 0.023.
**Figure S3.** Temporal validation of the multivariable prediction model for medical therapy failure in men with small‐volume benign prostatic hyperplasia. The model was derived on patients treated between 2015 and 2020 (n = 120, solid blue line) and temporally validated on patients treated between 2021 and 2025 (n = 81, dashed red line). Derivation AUC = 0.799 (95% CI 0.718–0.88); temporal validation AUC = 0.821 (95% CI 0.731–0.911).
**Figure S4.** Decision analysis curve showing the net clinical benefit of the full multivariable prediction model and selected single‐predictor models for predicting medical therapy failure in men with small‐volume benign prostatic hyperplasia (n = 201). The full model (green line) demonstrates the highest net benefit across the clinically relevant threshold probability range of 20–60%, outperforming single‐predictor models, the strategy of predicting all patients (dashed black line), and predicting none (dotted grey line).
**Figure S5.** Sensitivity analysis of the multivariable prediction model using only hard clinical endpoints (surgical intervention or acute urinary retention; 54 events). The model demonstrated stable discriminatory performance compared with the main analysis that included all failure events (symptom worsening, surgery, or acute urinary retention; 66 events). Solid blue line = main analysis (AUC 0.853, 95% CI 0.802–0.904); dashed red line = hard‐endpoint analysis (AUC 0.846, 95% CI 0.791–0.901).
**Table S1.** Multivariable Logistic Regression Coefficients and Integer Risk Score Derivation.

## Data Availability

The datasets generated and/or analysed during the current study are available from the corresponding author on reasonable request, subject to institutional approval.
