# Supplementary material for: Bedside risk score for medical therapy failure in small‐volume BPH: Temporal validation
Source: BJUI Compass. 2026 Apr 29;7(5):e70217. doi: 10.1002/bco2.70217 (PMC13125953; doi:10.1002/bco2.70217)
Supplement: Supplementary file 1 — Figure S1. Sensitivity analyses demonstrating robustness of independent predictors and model discrimination for predicting medical therapy failure in men with small prostate volume (<30 ml). Panel A shows the odds ratio for diabetes mellitus in the main model and monotherapy‐only sensitivity analysis. Panel B shows the area under the receiver operating characteristic curve (AUC) across three analytical subsets. Figure S2. Calibration plot of the final multivariable logistic regression model for predicting medical therapy failure in men with small‐volume benign prostatic hyperplasia (n = 201). The apparent (dotted) and bias‐corrected (solid) curves were obtained from 1000 bootstrap resamples. The dashed line represents perfect calibration (slope = 1, intercept = 0). Mean absolute error = 0.023. Figure S3. Temporal validation of the multivariable prediction model for medical therapy failure in men with small‐volume benign prostatic hyperplasia. The model was derived on patients treated between 2015 and 2020 (n = 120, solid blue line) and temporally validated on patients treated between 2021 and 2025 (n = 81, dashed red line). Derivation AUC = 0.799 (95% CI 0.718–0.88); temporal validation AUC = 0.821 (95% CI 0.731–0.911). Figure S4. Decision analysis curve showing the net clinical benefit of the full multivariable prediction model and selected single‐predictor models for predicting medical therapy failure in men with small‐volume benign prostatic hyperplasia (n = 201). The full model (green line) demonstrates the highest net benefit across the clinically relevant threshold probability range of 20–60%, outperforming single‐predictor models, the strategy of predicting all patients (dashed black line), and predicting none (dotted grey line). Figure S5. Sensitivity analysis of the multivariable prediction model using only hard clinical endpoints (surgical intervention or acute urinary retention; 54 events). The model demonstrated stable discriminatory performance compared [file BCO2-7-e70217-s001.docx]

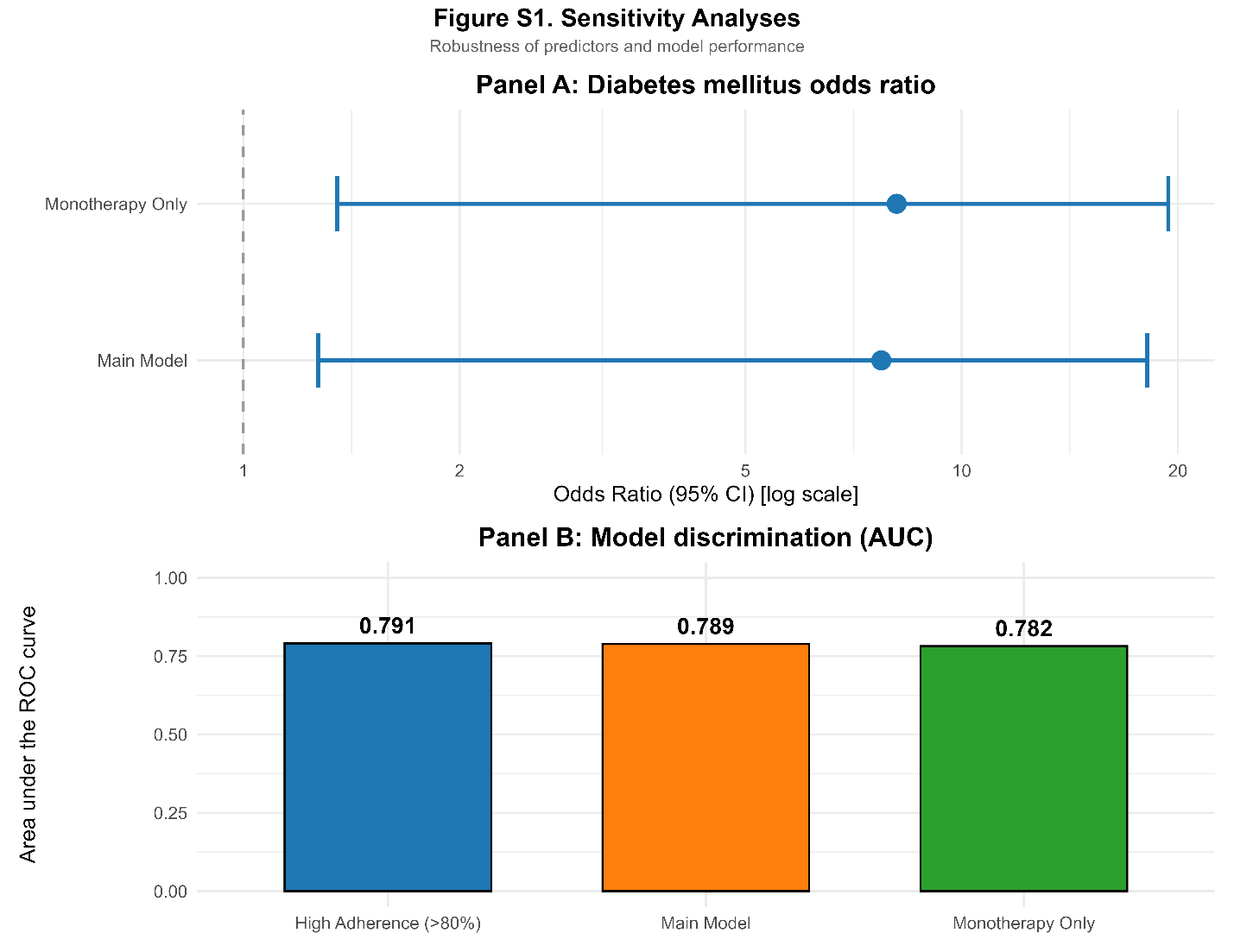


**Figure S1**. Sensitivity analyses demonstrating robustness of independent predictors and model discrimination for predicting medical therapy failure in men with small prostate volume (<30 mL). Panel A shows the odds ratio for diabetes mellitus in the main model and monotherapy-only sensitivity analysis. Panel B shows the area under the receiver operating characteristic curve (AUC) across three analytical subsets.


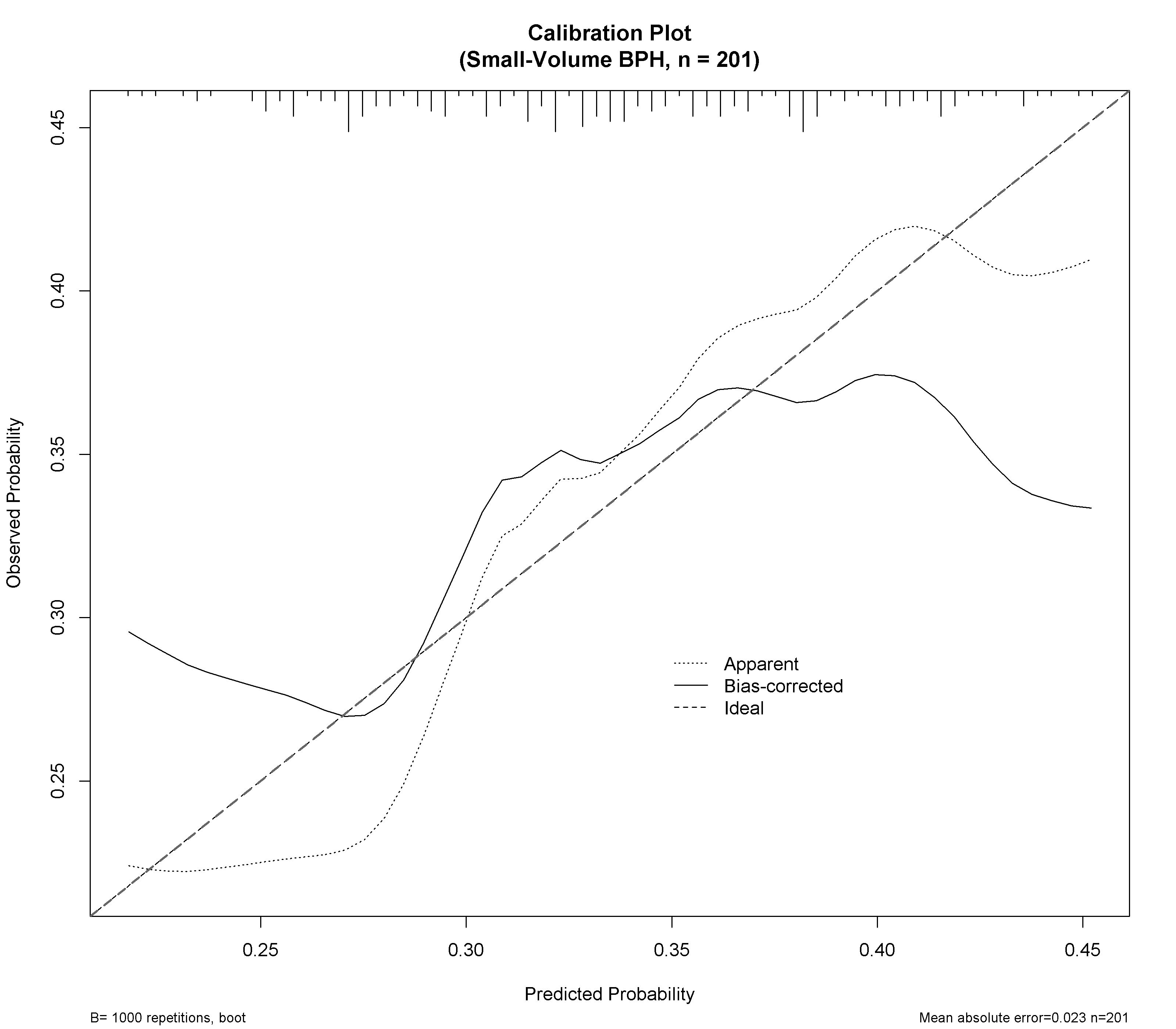


Figure S2. Calibration plot of the final multivariable logistic regression model for predicting medical therapy failure in men with small-volume benign prostatic hyperplasia (n = 201). The apparent (dotted) and bias-corrected (solid) curves were obtained from 1,000 bootstrap resamples. The dashed line represents perfect calibration (slope = 1, intercept = 0). Mean absolute error = 0.023.


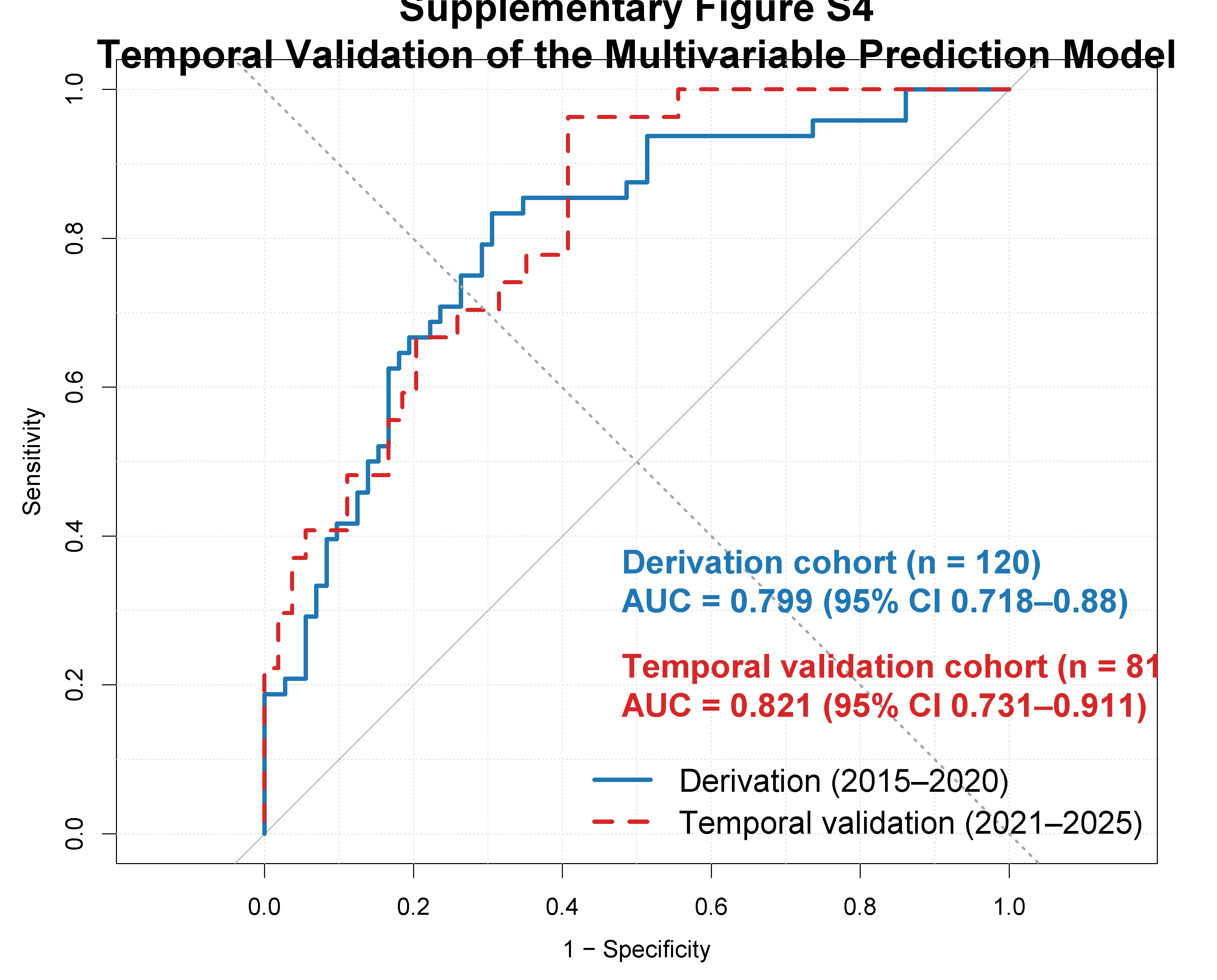


**Figure S3.** Temporal validation of the multivariable prediction model for medical therapy failure in men with small-volume benign prostatic hyperplasia. The model was derived on patients treated between 2015 and 2020 (n = 120, solid blue line) and temporally validated on patients treated between 2021 and 2025 (n = 81, dashed red line). Derivation AUC = 0.799 (95 % CI 0.718–0.88); temporal validation AUC = 0.821 (95 % CI 0.731–0.911).


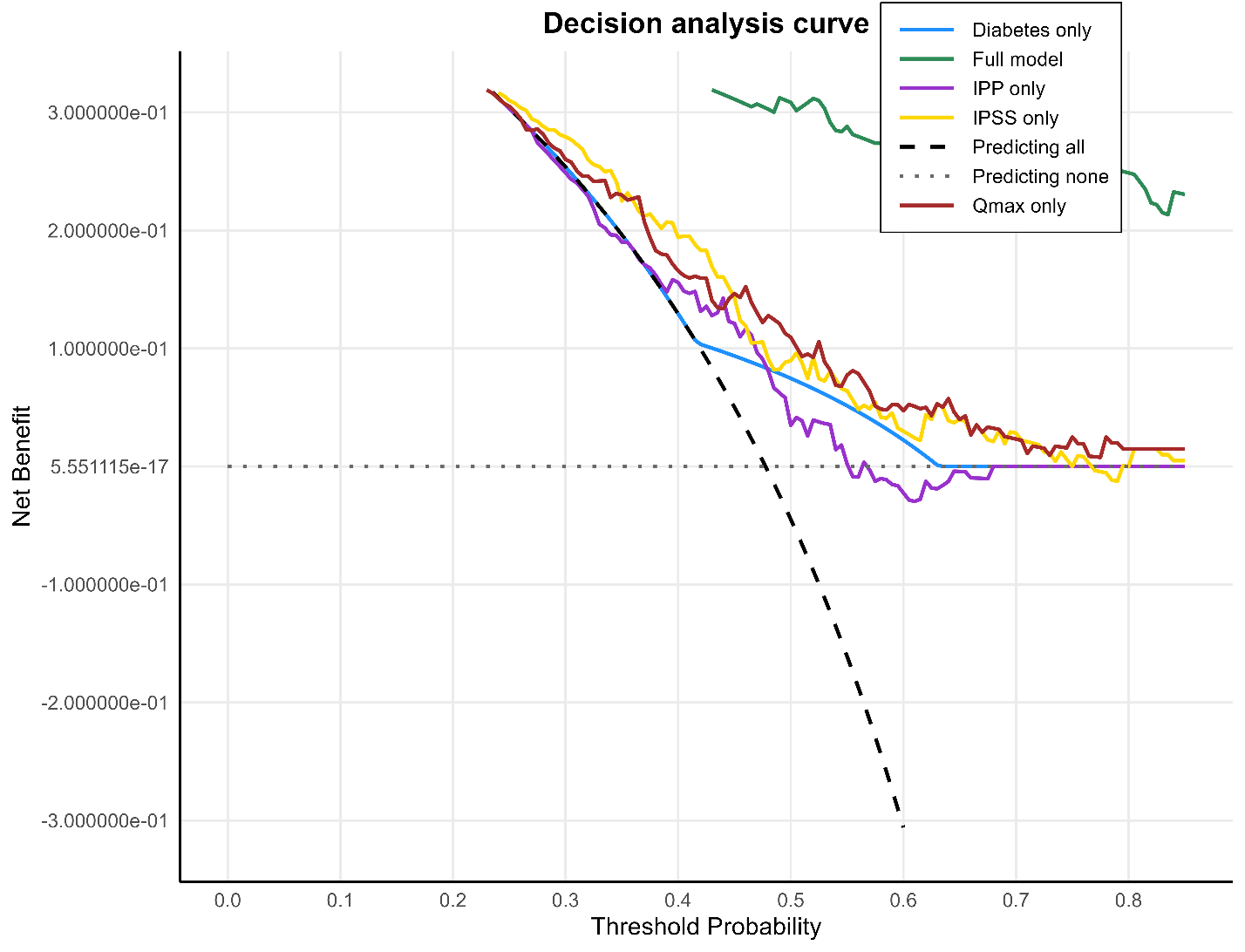


**Figure S4**. Decision analysis curve showing the net clinical benefit of the full multivariable prediction model and selected single-predictor models for predicting medical therapy failure in men with small-volume benign prostatic hyperplasia (n = 201). The full model (green line) demonstrates the highest net benefit across the clinically relevant threshold probability range of 20–60 %, outperforming single-predictor models, the strategy of predicting all patients (dashed black line), and predicting none (dotted grey line).


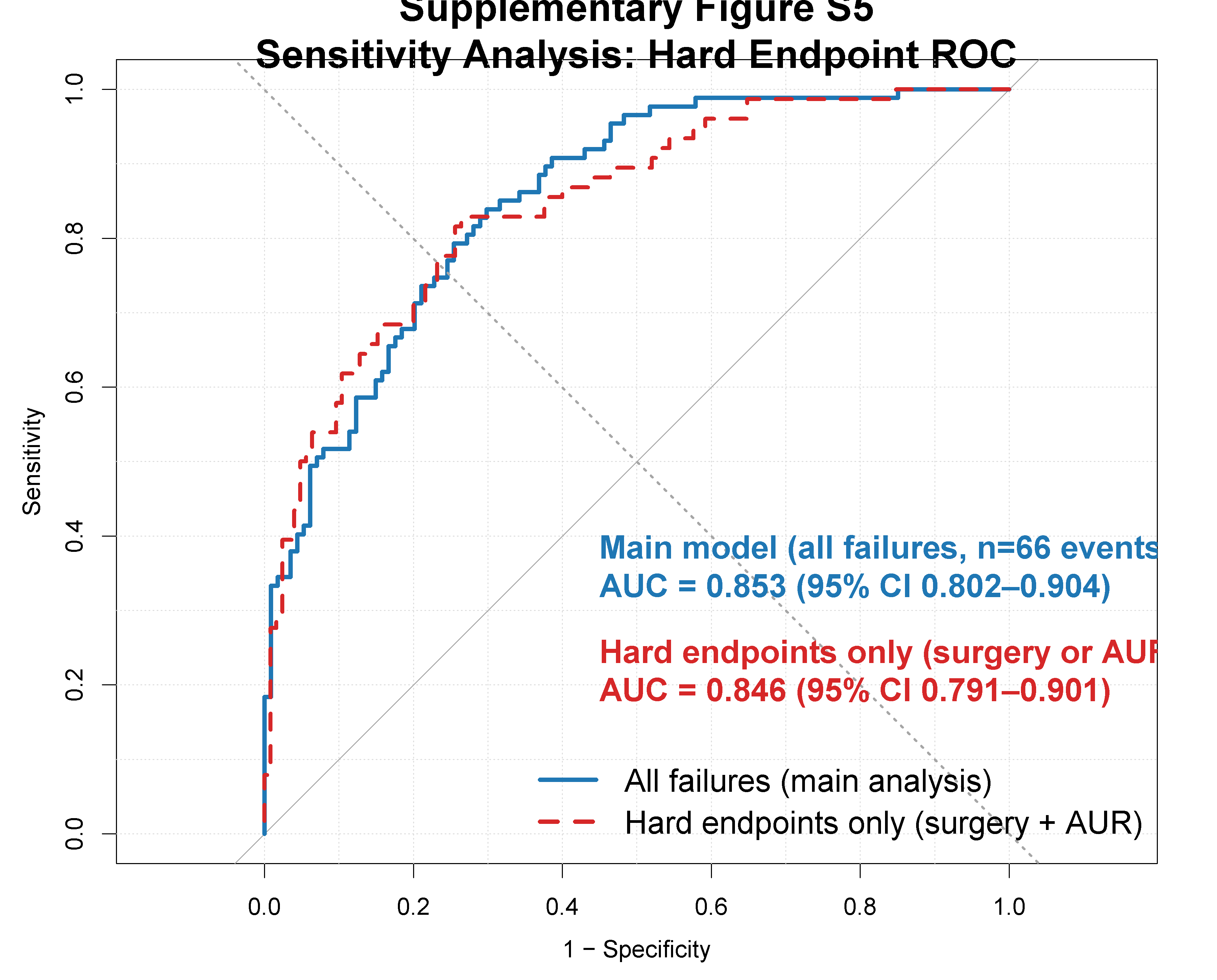


**Figure S5.** Sensitivity analysis of the multivariable prediction model using only hard clinical endpoints (surgical intervention or acute urinary retention; 54 events). The model demonstrated stable discriminatory performance compared with the main analysis that included all failure events (symptom worsening, surgery, or acute urinary retention; 66 events). Solid blue line = main analysis (AUC 0.853, 95 % CI 0.802–0.904); dashed red line = hard-endpoint analysis (AUC 0.846, 95 % CI 0.791–0.901).

**Supplementary Table S1:** Multivariable Logistic Regression Coefficients and Integer Risk Score Derivation

| **Predictor** | **β coefficient** | **OR (95 % CI)** | **Points assigned** | **Rationale / Cut-off used** |
| --- | --- | --- | --- | --- |
| Diabetes mellitus (present) | 2.045 | 7.73 (2.71–22.05) | 4 | Strongest predictor |
| IPSS ≥20 | 0.293 | 1.34 per point | 3 | Severe category |
| IPP ≥8 mm | 0.322 | 1.38 per mm | 2 | Youden index + clinical practicality |
| Qmax <8 mL/s | −0.371 | 0.69 per mL/s | 2 | Severe obstruction threshold |
| PVR ≥100 mL | 0.039 | 1.04 per mL | 2 | Clinically meaningful residual |

β coefficients are from the final multivariable model fitted on the derivation cohort. Points were scaled proportionally and rounded for bedside use
